# Setting up and maintaining a dedicated student-led peer-assisted learning society: our experience and recommendations

**DOI:** 10.15694/mep.2017.000165

**Published:** 2017-09-19

**Authors:** Kristen Davies, Christiana Rousseva, Huzaifah Khojani, Natalia Kyrtata, Fatimah Khoda, Joanna Heyworth

**Affiliations:** 1 Lancaster University; 2 University Hospitals of Morecambe Bay

**Keywords:** peer-assisted learning

## Abstract

This article was migrated. The article was marked as recommended.

Peer-assisted learning (PAL) is becoming increasingly popular within medical education, reflected by the amount of literature on the subject. There are numerous benefits of PAL for both teachers, students and faculty. At Lancaster Medical School, we decided to first investigate whether students wanted a student-led PAL society. Following the results, we set up the Lancaster University Peer-Assisted Learning Society (LUPALS) in 2013. Since its foundation, LUPALS has successfully provided over 100 teaching sessions to medical students at Lancaster Medical School. We have highlighted the important aspects of setting up our PAL society with reference to the evidence base and provided recommendations for others who are considering creating their own PAL society at their institution. We conclude that setting up LUPALS has been a successful venture and should act as encouragement for others who wish to do the same.

## Introduction

Peer-assisted learning (PAL) is commonly used throughout all areas of healthcare education and is becoming increasingly popular within medical education. Numerous authors have presented reasons to practice or engage with PAL ( Ten Cate and Durning, 2007, Burgess et al., 2014, Seifert et al., 2016, Menezes et al., 2016, Furmedge et al., 2014). PAL can help alleviate faculty teaching burden for teachers and doctors within the medical school, as well as providing teaching experience for medical students ( Ten Cate and Durning, 2007). Further benefits of PAL include providing role models for students and developing the ability to teach and convey information clearly - key requirements of the General Medical Council’s (GMC) ‘Outcomes for Graduates’ ( General Medical Council, 2015).

Lancaster Medical School (LMS) is a medical school in Lancaster, UK, whose first cohort graduated with Lancaster medical degrees in 2017. Within LMS there were popular, yet sporadic, peer-lead teaching sessions from the senior students who had previously expressed the desire to teach their peers. They were, however, unable to do so due regularly to the lack of a dedicated student body to facilitate such teaching. We hypothesised that a dedicated PAL society could organize teaching sessions for medical students, increase the amount guidance available and increase the provision of learning resources available to students, which have been suggested to reduce student stress levels ( Moffat et al., 2004).

We firstly wanted to determine whether students at a small undergraduate medical school wanted a peer-assisted learning society. Based on those results, we then created the Lancaster University Peer-Assisted Learning Society (LUPALS) and helped organize and deliver teaching sessions to medical students.

## Addressing the need for a PAL Society

**Figure 1.**
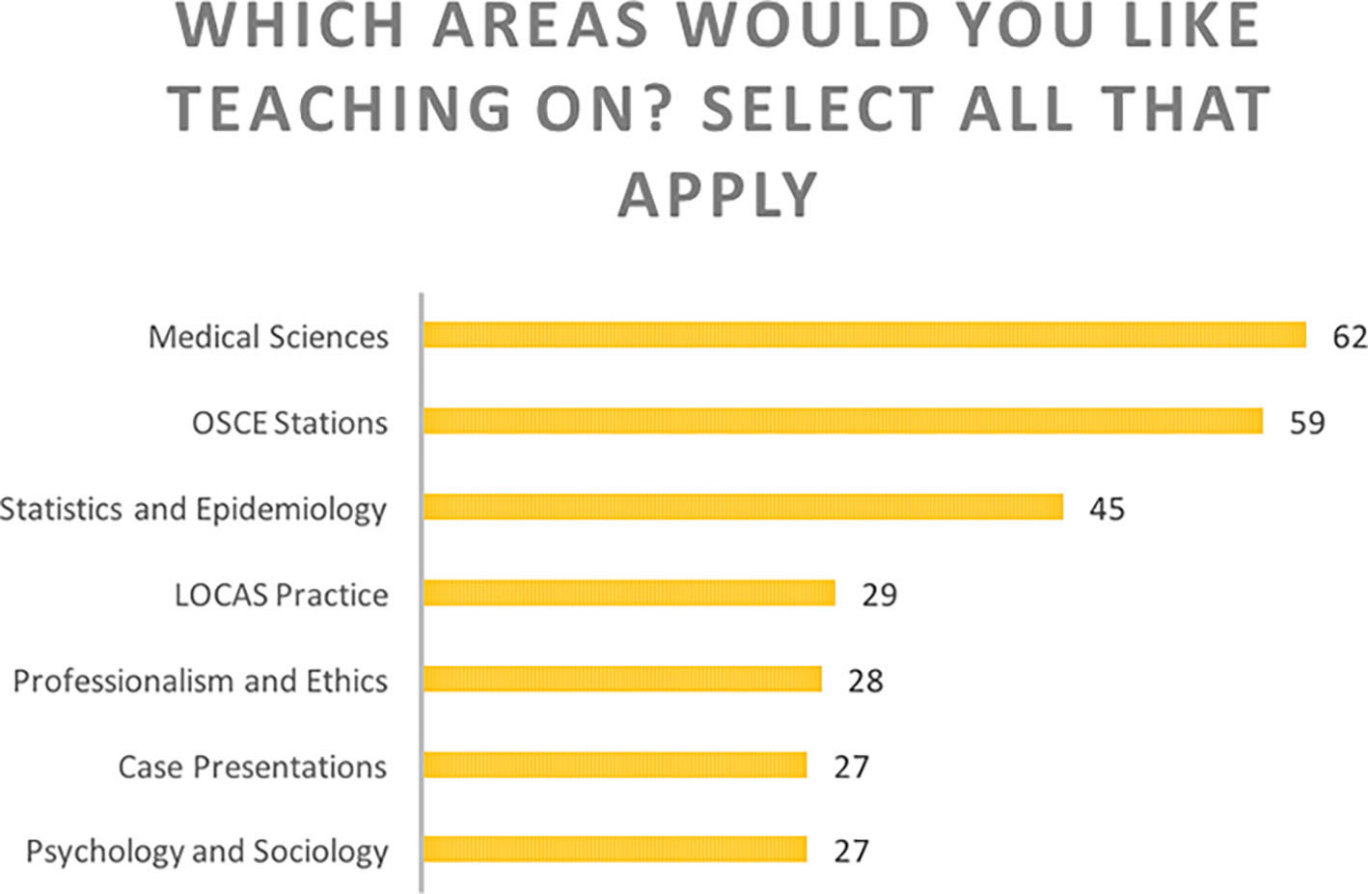
Examples of our initial survey results: What areas would you like teaching on? (n=71). Participants could select as many areas of the curriculum which they desired teaching on. The areas are ordered from the most requested (medical sciences) to least requested (psychology and sociology). The four core areas of our PBL curriculum were listed in addition to clinical practice in the form of OSCE Stations, LOCAS and Case Presentations. LOCAS is the Liverpool Objective Clinical Assessment which was used as the clinical assessment for fourth year medical students. Percentages are displayed.

To initially gauge the level of interest that we might receive by setting up LUPALS, we sent out a short informal survey to all undergraduate students at Lancaster Medical School. An example of our survey results are shown in Figure 1. Seventy-one (29%) students responded to the initial survey. The majority of respondents (90%) expressed that they would be interested in being taught from sessions hosted by a PAL society. Free-text comments included:

*“[LUPALS] gives a unique opportunity to begin teaching in medical school, and provides even more teaching for lower years, at an accurate level for their abilities.”*

*“We get taught every week by intercalating students while on the medical rotation and it is great. I’d have loved it to be every week throughout the surgery rotation too.”*

Whilst the majority of respondents felt enthusiastic about LUPALS, many also expressed concerns:

*“The teaching materials/presentations would have to be to a high standard, and the teaching students would have to be enthusiastic about their topics.”*

*“A potential pitfall is if someone teaches ineffectively, students may be less inclined to attend the sessions. The teaching has to be thought through and delivered by someone with some experience in this area.”*

Following our positive response, we also discussed LUPALS with the Director of PBL and the Director of Medical Studies at the medical school, who were both enthusiastic about LUPALS. Important issues were raised, however, including the potential undermining of scheduled lectures and of the curriculum style of problem-based learning (PBL), where students use their own resources to learn information rather than from didactic lectures. To reduce the risk of undermining the PBL curriculum style and the teaching that students already receive, it was agreed that we would only cover topics after the end of the corresponding module.

To help us set up an effective society, we attended two conferences. The ‘Peer Assisted Learning in Medical Education’ (PALME) conference, at Sheffield Medical School, allowed us to gain an insight into how such a society is effectively functioning elsewhere. We were able to discuss the successes and challenges that PALME had faced in their first few years, as well as to pick up hints and tips for our society. At the Junior Association for the Study of Medical Education (JASME) workshop at Hull York Medical School, we discussed strategies for effective peer-teaching with professionals in medical education. PALME ultimately helped us to design our committee, whilst JASME formed the basis for our first teacher training.

Through the online survey, discussions with staff and attending the conferences, we identified three potential pitfalls of the society. Firstly, there is a risk that students in older years would deliver incorrect or out-of-date information. Secondly, if the peer-teachers lack effective communication skills, there is a risk that less experienced students may become more confused about a topic. Finally, there is a danger that students would receive revision sessions based on what the peer-teachers need to revise, not what the less experienced students most struggle with. These dangers persist in the society today, however we aim to ameliorate them through the way that the society is structured and through regular teacher training.

***Recommendation #1*:** Engaging with faculty members of the Medical School not only helps develop societies such as LUPALS but also identify potential pitfalls and areas of consideration.

***Recommendation #2*:** Collaboration and engaging with medical students can identify areas where improvement is needed which can help the society understand its place within the medical education curriculum.

## Developing the society

Having identified the needs for LUPALS and its place within the medical education curriculum, we need to address the aforementioned potential pitfalls. Two sides of the society were recognised as needing development; the societal committee and the peer-teachers who would be delivering the sessions.

### The committee

All roles were devised to ensure the longevity of the society, which meant that many long term considerations were taken. These positions would result in a very diverse group of individuals running the society; students from all year groups within the medical school with varying interests. In order to mitigate the relatively large responsibility and workload for each of the roles, candidates for the positions were encouraged to nominate themselves in pairs.

In addition to the President, the majority of the committee was formed by a position known as the coordinator. Each year group, from years one to four, has its own coordinator (or pair of coordinators) who is responsible for organising all teaching for their respective year group. Tasks involve devising a curriculum that would be relevant for the cohort, finding more senior students to teach these topics and then arranging an appropriate time and place for the teaching session to take place so that the maximum number of students could attend. A year group’s coordinator must be a student that was currently in the year group above, as it means that they were in a better position of understanding the students’ learning needs, having recently experienced it themselves. Considering one of the pitfalls identified was the risk that peer-teachers may teach what they needed to revise, rather than what students wanted, co-ordinators regularly reviewed the requests of their respective year groups in order to meet the learning needs of their students. Interpersonal skills were of utmost importance due to the large number of students that they would need to liaise with in order to recruit teachers.

Involving first-year students involved was considered crucial. Involving them with the society early could increase their interest in medical education and potentially lead them to teach through the society in the future. For this reason, the role of outreach officer was created as first-year students did not have a less experienced year group whom they could teach. The role centered around widening participation to medicine by building relationships with local schools and colleges. This role developed into a collaboration with the Medical School’s Director of Admissions.

### The peer-teachers

Our initial survey had suggested that students wanted to get involved in teaching medical students but how we regulated our teachers was a matter of importance. The issue of quality assurance has previously been discussed in the literature ( Iwata and Furmedge, 2016) and highlights that peer-teachers must have training in teaching so that students’ learning experiences are not compromised. Indeed, PAL has been reported to be most effective when clear standards or guidelines are provided for peer-teachers ( Tai et al., 2016).

It was decided that to become a peer-teacher, students would have to attend a ‘Teacher Training’ evening held at the medical school. The teaching training sessions were established to train peer-teachers in a number of areas including practical teaching skills and presentations tips. The sessions of effective teaching skills were aimed to address the potential pitfall of ineffective communications skills that teachers may have. The teacher training sessions were delivered by the society with speakers including academic doctors from the medical school and clinical doctors from the local hospital trust.

In order to ensure the quality of the teaching sessions, it was decided that all sessions must go through a peer-review process by an approved peer-teacher, who must a) have attended a teacher training evening and b) be in a year group at least one year above that which the session they are reviewing is for. By ensuring a peer-review process, we aimed to address the potential pitfall of delivering incorrect information to students. The peer-review process could take place either as a live-run through or a detailed lesson plan with all information which was to be delivered.

Following the creation, peer-review and delivery of a LUPALS teaching session, peer-teachers received feedback from paper feedback forms containing five questions and free text which they could keep for their portfolio. A survey of UK anatomists found this to be this method of certification was considered to be the most suitable for peer-teachers (38%) ( Stephens et al., 2016b).

### Accreditation and results from the foundation year

To formally setup the society, our plan was presented to the Lancaster University Student’s Union (LUSU) and interested medical students. The society formally started in the academic year of 2013/14.

The society ran 17 sessions between 24/03/13 and 17/10/14 for medical students from years 1-4. The first teaching training event occurred on the 24 ^th^ March 2014 and attracted 58 students. Positive feedback from teaching sessions and an average attendance of 25/50 (50%) of students in each year group encouraged the society to continue. An online drive was also created where material was accessible to all committee members, and a separate folder with all teaching material was shared with all students which was available to them for revision purposes.

***Recommendation #3***: Students wanting to create a PAL society should consider methods of quality assurance to ensure that correct information is being taught to students.

***Recommendation #4***: Successful ways of incentivizing potential peer-teachers includes; networking opportunities, practical teaching advice and evidence for their C.V. and portfolios.

***Recommendation #5***: Incorporating an outreach officer for widening participation encourages more junior medical students to engage with our PAL society.

## Building on the foundation year; 2014/15 and 2015/16.

LUPALS’s first year was largely successful. Feedback from students was very positive and the number of students wishing to be peer-teachers was increasing. Changes to the society mainly aimed to make the society more effective in delivering teaching sessions. Over the next two academic years, the number of LUPALS sessions increased to 48 and 51, respectively.

### Changes to the committee structure

It was evident from the first year that a large committee was not conducive to the committee’s productivity. Roles were combined to give committee members more responsibility and the opportunity to play a bigger role in the society’s decision-making. As a result, attendance to committee meetings was increased, misunderstandings about committee roles were reduced and more teaching sessions were organised by the end of the year.

### Teacher training

The date of the teacher training session was set to suit the majority of students. However, with the variable timetable of medical students, there was still a large proportion that was unable to attend. Since teacher training is a requirement for all students wishing to teach, a second session was arranged later in the year. Sessions were shortened to maintain attention and interest and a detailed information pack was handed to all attendants, minimising the information mentioned during the training session.

The training activity previously consisted of a medically-related topic. More experienced students found the task relatively easy, whereas less experienced students heavily relied on the information that was handed to them during the session. In order to give everyone an equal opportunity to test their teaching skills, two non-medically-related topics were introduced: “how aeroplanes fly” as a theoretical topic and “how to rewire a plug” as a more practical topic. Students seemed to enjoy the activity and came up with more ideas to improve a lesson, such as introducing a real-life model or drawing diagrams.

### Teaching sessions

We found that the main barrier to organising teaching sessions was the peer-review. Students found it challenging to get their work peer-reviewed, signed and handed back to the committee in time for their lesson. Standardised, online forms were subsequently developed which made the process much quicker, more accessible and easier for the committee to track all past teaching sessions.

In terms of the teaching material itself, topics were previously too broad, involving whole body systems such as “the neurological system” or “respiratory pathology”. This was particularly challenging for teachers who had to deliver short sessions on large topics, and students frequently found the sessions overloaded with information. Social media groups were created to directly ask students which specific topics they found most challenging, and subsequent sessions were more targeted and focused. Students would often request revision sessions on large topics, especially before exams, but this approach was largely successful and allowed teachers to go into more depth on particularly challenging topics and have more time to answer questions.

The initial survey results ( Figure 1) showed that students predominantly wanted teaching sessions around medical sciences topics (such as anatomy, pathology etc.) but a large proportion also wanted practice for Objective Structured Clinical Examinations (OSCEs). To meet this demand, OSCE teaching sessions were organised. In each session, student-teachers would demonstrate a particular clinical skill, focusing on the challenging aspects or parts which students usually struggle with. Students were then given the opportunity to practice under supervision. Unlike previous student-lead clinical skills sessions which took the form of a mock OSCE, organized by the Medical Society (MedSoc), this session gave students the opportunity to ask more questions and lean about the details which would help them excel during their exams. This way, LUPALS focused on the teaching of the clinical skills, whereas MedSoc gave the opportunity to demonstrate their skills.

***Recommendation #6***: Collaboration with other medical societies can provide students with a well-rounded learning experience where they can both learn and demonstrate their knowledge.

***Recommendation #7***: Online documentation of peer-review and feedback forms can useful means of documentation for both students and teachers.

***Recommendation #8***: PAL societies can maximize student interest and attendance by engaging with students to see what they need and by offering sessions with targeted learning outcomes so students know what to expect.

## Moving forward: 2016/2017 and beyond

### Changes to LUPALS

2016/17 saw the introduction of crash course weekends for year 3 and year 4 medical students. External revision weekends are popular among medical students but due to their costly nature, not all students are able to attend. As a committee, we collectively decided that it would be beneficial for students if we ran a similar course at the university campus. During the planning for this weekend, we researched which subjects would be most beneficial by asking the year group in question, as well as seeking advice from upper year students.

After careful planning, we ran two revision weekends that consisted of a two-day course split into a series of tutorials on a variety of subjects, with a quiz at the end to consolidate all that was learnt on the weekend. Handouts were provided, as feedback highlighted they were found to be useful revision aids. The courses were a great opportunity not only for the year group in question, but also for the more mature years who were able to get ample teaching experience. Feedback on both weekends indicated that the courses were well attended and received.

In previous years, we began to provide some clinical skills teaching as part of LUPALS, as students in the first two years of medical school can find this particularly challenging. We found that putting on clinical teaching sessions was a great way for students to ask questions and practice in a supportive environment as well as building their confidence for the clinical setting. In particular, we provided extra history-taking for the first year students who are exposed to this skill for the first time and third year students, who can find histories tailored to specific specialties, such as psychiatry and obstetrics, difficult to grasp.

### Challenges faced

Although this has been a very successful year, we have faced some difficulties in running the society. We found that teaching sessions were not as well received in some years, with consistently low turnout rates. This had a profound effect on teacher morale and led to an unwillingness to continue teaching. Other challenges we faced were getting teaching sessions for senior students earlier on in the academic year due to work load pressures.

## Future work

To develop the society further, we feel that it would be useful to implement revision weekends for junior medical students. It may also be useful to try and arrange a system through which foundation year doctors at the hospital could teach the senior medical students through LUPALS, providing them with more teaching sessions earlier on in the academic year.

Whilst we have ensured that each peer-teacher is supported prior to a session by having their content peer-reviewed, we have not provided a formal opportunity for peer-teachers to reflect on their practice. Reflection is an important aspect of learning and perhaps by providing a session where peer-teachers can reflect on their feedback, individually and within a group, they can identify areas for improvement and discuss with their colleagues. Development of such skills are critically important for a career in medicine but it also helps encourage our peer-teachers to develop a self-monitoring approach towards developing their own teaching practice ( Kolb, 2014). This will potentially provide an additional layer of quality assurance for teaching sessions provided through LUPALS.

Most importantly, we must look for ways to maintain the interest of our peer-teachers. By requiring peer-teachers to attend a teacher training event, in addition to the requirement that each lesson needs to be peer-reviewed by an appropriately experienced student, we are potentially discouraging students from wanting to become peer-teachers. Whilst we maintain that these steps are necessarily to provide quality sessions to medical students, we acknowledge that these areas require consideration. One way of maintaining student interest in LUPALS may be through involving peer-teachers in research. LUPALS can allow students the opportunity to collect and analyse data with support from the faculty ( Stephens et al., 2016a). By offering long-term academic rewards, such as through an analysis of longitudinal data, peer-teachers may be motivated to be involved with PAL for longer periods and it also provides an extra incentive for students to sign up as peer-teachers initially ( Bovil et al., 2014)

***Recommendation #9***: PAL societies should consider running revision weekends for students in addition to regular revision lectures.

***Recommendation #10***: Involving peer-teachers in research could help keep them interested in engaging with a PAL society.

## Conclusion

Peer-assisted learning has well known advantages within medical education and our society has enabled students to formally teach their peers. The current article makes the case for why a student-led PAL society can be beneficial to undergraduates, in addition to the problems we faced and our rationale for our recommendations.

Considering our venture into PAL was a student-led initiative, we thought that an account of our positive and negative outcomes, along with our recommendations, would be beneficial to students who wish to engage with PAL or are already involved with PAL at their medical school. Similarly, our account may provide informative to academic members of the medical school, as they can witness how we developed a medical education curriculum to complement the one provided to us by our institution. Whilst we have given our opinion of how LUPALS has progressed and grown we also need to formally collect feedback from students to review LUPALS and this remains a project for the current academic year.

Setting up a PAL society in our medical school has been a successful and positive process and should act as encouragement for other medical schools to develop a PAL society. Students who are interested in developing a PAL society should first determine the level of interest, consider how they will approve people to teach (through training and reviewing) and ways which they can generate feedback in ways which are quick and suitable for both students and teachers alike.

## Take Home Messages

## Notes On Contributors

Kristen Davies is a final-year medical student at Lancaster Medical School, Lancaster, UK. He is a co-founder and first year co-president of the Lancaster University Peer-Assisted Learning Society. He has previously been awarded the Clegg Scholarship in Medical Education from the BMJ.

Christiana Rousseva is a final-year medical student at Lancaster University, Lancaster, UK. She is a co-founder and first-year co-president of LUPALS. She has an interest in medical education and has previously presented research on learning environments internationally at the International Association of Medical Education (AMEE) conference.

Huzaifah Khojani is a foundation doctor at the Royal Lancaster Infirmary, Lancaster, UK. He is a co-founder and first-year co-president of LUPALS.

Natalia Kyrtata is a final-year medical student at Lancaster Medical School, Lancaster, UK. She was a member of the founding committee of the Lancaster University Peer-Assisted Learning Society and president of the society for two consecutive years.

Fatimah Khoda is a fourth-year medical student at Lancaster Medical School, Lancaster, UK. She was co-president of LUPALS during the 2016/17 academic year and is currently working towards completing the NHS Leadership Academy’s Edward Jenner Programme.

Joanna Heyworth is a fourth-year medical student at Lancaster Medical School, Lancaster, UK. She was co-president of LUPALS during the 2016/17 academic year. She has been an active member in both teaching and the committee from 2014.
