# Supplementary material for: Setting up and maintaining a dedicated student-led peer-assisted learning society: our experience and recommendations
Source: MedEdPublish (2016). 2017 Sep 19;6:165. [Version 1] doi: 10.15694/mep.2017.000165 (PMC10885295; doi:10.15694/mep.2017.000165)
Supplement: Peer review history

# Reviewer report: Reviewer response for version 1

**DOI:** 10.21956/mep.19337.r28244

**Published:** 2017-10-23

**Authors:** Ebbe Thinggaard

**Affiliations:** 1 Copenhagen Academy for Medical Education and Simulation

This review has been migrated. The reviewer awarded 4 stars out of 5 The paper describes the authors experience creating a student-led peer-assisted learning society. The steps in setting up such a society are described in a systematic manner addressing the student’s needs, the formation of the society, the setting up and running of the society as well as the future perspectives. The paper addresses the challenges in setting up a peer-assisted learning society as well as how these challenges can be overcome. Each section also includes useful recommendations for the readers, on how to set up a peer-assisted learning society. In conclusion, the paper is a highly valuable contribution and should be read by anyone interested in peer-assisted learning and particular readers considering starting their own peer-assisted learning society.

Reviewer Expertise:

NA

No decision status is available

# Reviewer report: Reviewer response for version 1

**DOI:** 10.21956/mep.19337.r28243

**Published:** 2017-09-24

**Authors:** Trevor Gibbs

**Affiliations:** 1 AMEE

This review has been migrated. The reviewer awarded 4 stars out of 5 A very well written and well structured paper regarding an increasingly used approach to teaching. I think that the use of a student learning society was excellent and I particularly liked the evolving nature of the activity, the positives and the negatives. A very important paper to read if interested in this area; I will look forward to seeing how this develops

Reviewer Expertise:

NA

No decision status is available
